# Shotgun and Hi-C Sequencing Datasets for Binning Wheat Rhizosphere Microbiome

**DOI:** 10.1038/s41597-025-04651-3

**Published:** 2025-03-01

**Authors:** Roshan Regmi, Jonathan Anderson, Lauren Burgess, Hayley Mangelson, Ivan Liachko, Gupta Vadakattu

**Affiliations:** 1 Microbiomes for one Systems health (MOSH), CSIRO, Adelaide, Australia; 2 Agriculture and Food, CSIRO, Urrbrae, South Australia; 3 Agriculture and Food, CSIRO, Floreat, Western Australia; 4 The UWA Institute of Agriculture, University of Western Australia, Crawley, Western Australia; 5 Phase Genomics, Seattle, WA USA

**Keywords:** Data integration, Genome informatics

## Abstract

Binning is a crucial process in metagenomics studies, where sequenced reads are combined to form longer contigs and assigned to individual genomes. Conventional methods, such as shotgun binning, rely on similarity measurements and abundance profiles across multiple samples. However, cost constraints for sequencing and limited sample collection capacity hinder their effectiveness. High-throughput chromosome conformation capture (Hi-C), a DNA proximity ligation technique, has been adapted to accurately bin metagenome-assembled genomes (MAGs) from a single sample, addressing challenges like chimeric MAGs. In this study, we generated over 190 Gb of metagenomic data from wheat rhizospheres grown in two highly calcareous soils of South Australian region and compared conventional and Hi-C binning methods. Two shotgun metagenomes and Hi-C libraries were generated, assembling 1089 shotgun MAGs across 39 bacterial and one archaeal taxon, including 94 Hi-C based bins. Binning performed using only short read sequences was prone to high contamination, while the addition of Hi-C binning improved MAG quality and identified mobile element-host-infection interaction. This dataset provides important tools for studying microbial communities in wheat rhizosphere soils.

## Background & Summary

The rhizosphere is one of the most complex ecosystems, where numerous microorganisms interact with the roots of host plants within a small region (2-3 mm from the root surface) of soil surrounding the roots^1^. Rhizosphere microbiomes are key to maintaining plant essential functions by influencing their physiological processes, health and development. Understanding complex interactions among microbial species in the rhizosphere allows deeper understanding of their mechanistic and mutualistic functions^2^. This information can be used to manipulate rhizosphere community formation and optimise microbiota-mediated functions to improve plant growth and yield. Advancement of high-throughput sequencing platforms applied to metagenomics has improved our understanding of the metabolic potential of microbiomes. However, an understanding of the mechanistic functions driven by plant-microbe interaction remains elusive due to the complexity of interactions among soil microorganisms with plants and other environments. In metagenomics studies, binning is one of the fundamental processes where sequenced reads generated from the community are stitched together to form longer contigs and these contigs are assigned to an individual genome^3^. In conventional shotgun binning, reads are assembled into a genome based on contig similarity measurements from GC-content^4^, tetra-mer composition^5^ and/or co-abundance feature of the contigs across multiple samples^6,7^. Although abundance profiles across multiple samples have been useful in the discovery of novel microorganisms^8^, the requirement of a sufficient number of samples to obtain reliable associations between contigs may not be feasible due to cost constraints for sequencing and limited sample collection capacity. These limitations reduce the effectiveness of conventional binning methods. In contrast, high-throughput chromosome conformation capture (Hi-C) is a DNA proximity ligation technique, originally developed to investigate the 3D structure of individual genomes, has been adapted to accurately retrieve metagenome-assembled genomes (MAGs) from a single sample without the requirement of high molecular weight DNA extraction. The Hi-C technique produces millions of paired-ends reads that link DNA fragments found in proximity within unlysed cells. Hi-C data combined with traditional shotgun sequencing can recover high-quality MAGs from a single sample^9,10^. One of the challenges in conventional metagenome binning is the creation of chimeric MAGs where DNA fragments from different organisms are combined. Hi-C data addresses this challenge by providing information about sequences that were physically proximal inside of a cell membrane, thus minimizing MAG contamination.

The rhizosphere, where microbes and plants coexist in a carbon-rich environment, is a hotspot for mobile genetic element (MGE) transfer. It has been proposed that ancient MGE translocations drove the evolution of both microbes and plants^11^. Understanding this process provides fundamental knowledge to our understanding of microbiome communities mediated by locally adaptive genes rather than species^12^. While movement of mobile elements occurs for self-replication and transmission, these elements often carry host genes to which they have become linked. Sometimes, such events are so pronounced as to impact host fitness. For example, transfer of conjugative genes with linked nodulation and nitrogen fixation genes converts non-symbiotic bacteria to symbiotic in a single step^13,14^. Mobile elements have also been shown to transfer antimicrobial resistance and heavy metal resistance genes^15,16^. Therefore, investigation of MGE transfer in the rhizosphere provides useful information for studying pathogenic/beneficial microbe-plant interactions. Hi-C is a useful technique to assign mobile elements to their hosts because these DNA elements (including infectious phage) can be found physically touching the host genome inside of the cell membrane, therefore providing opportunities to capture the relationship^17^. This information allows us to better understand the complex role of mobile elements in the soil rhizosphere, including tracing elusive horizontal gene transfer events.

Highly calcareous soils are limited by several physico-chemical constraints for crop growth and pasture productivity. Identification of the key factors constraining crop production on these soils is essential to understanding how these factors affect microbial processes, functions and crop growth. One way to address the functional potential of these soils is by understanding the metabolic profile of microorganisms in these soils through next generation sequencing such as metagenomics. Calcareous soils are widely distributed in semi-arid, arid and sub-tropical regions around the world with more than 30% of global soils classified as calcareous^18^. There are 1.5 M ha of highly calcareous (>15% CaCO_3_ w/w in the topsoil) soils in the Southern cropping region with a further 1.4 M ha of moderately calcareous soils^19^. Key sub-regions within South Australia that have a high proportion of highly calcareous soils include Western Eyre Peninsula and Lower Yorke Peninsula while moderately calcareous soils are important to the crop producing areas of central Eyre Peninsula, Upper Yorke Peninsula, the Murray Mallee and the Lower Southeast. Here, we compare traditional shotgun metagenome assembly approaches with Hi-C binned genomes from two calcarosols soils collected from Southern Australian cropping regions i.e., Eyre Peninsula (Poochera) and Mallee (Avon), hereafter named as S1 and S2, respectively. The results revealed the successful generation of Hi-C libraries for S1 with improved quality of recovered MAGs. However, the ability of Hi-C data to improve binning and mitigate contamination may be limited by multiple factors in some samples as Hi-C did not work effectively in S2 soil. Utilizing Hi-C for complex soil samples is still at a very early stage and the variability in soil sample quality and biomass is a well-known challenge in the field. It can be difficult to concentrate sufficient biomass from complex soil, which is an issue that affects most sequencing approaches and is often hard to predict in advance. For each sample, ProxiMeta results in enough reads but variability in sample quality leads to differences in genome recovery and the overall quality of the final outcomes, which is also evident from assembly statistics that for S2 sample only 0.44 percentage of the assembly length are represented in clusters (Table 1). In this case, it’s likely that greater biomass concentration and consequently more microbial DNA would have enabled deeper sequencing, allowing for the characterization of more than just the highest-abundance species in low MAGs recovered sample (S1). When biomass is low, both data quality and sequencing depth can be compromised. Since all data from this study are publicly available, this manuscript provides caution for prospective Hi-C users and presents an opportunity for further investigation into the specific factors impacting the performance of Hi-C for assembling metagenomes from complex samples such as soils.

**Table 1:** Hi-C based assembly information.

Assembly features	Poochera (S1)	Avon (S2)
**Assembly length**	1,450,169,505	3,353,096,377
**Number of contigs total**	767,913	1,955,345
**Number of contigs in clusters**	24,204	3,275
**Percentage assembly length in clusters**	10.67	0.44
**Percentage contigs in clusters**	3.15	0.17
**Total bins**	88	8
**Complete clusters (>95% complete, <10% contamination)**	6	0
**Excellent clusters (>90% complete, <10% contamination)**	8	0
**Good clusters (>70% complete, <10% contamination)**	15	1
**Reasonable clusters (>50% complete, <10% contamination)**	26	1

## Methods

### Sample collection and sequencing

Topsoil 0–10 cm were collected from S1 and S2 sites, at S32 43.335, E134 50.32224 and S34 13.981, E138 18.586, respectively. Soil weighing one kilogram was placed into 1.5 L pots. Soils were inoculated with *Rhizoctonia solani Ag8* to mimic the rhizoctonia incidence in the South Australian region. The pots were then kept at 15 °C for a week to allow for incubation. To maintain the desired moisture levels of 12% w/w for S2 and 16% w/w for S1 soil, the pots were watered twice a week. Following a 7-day incubation period, four seeds were planted in each pot, and the soil surface was covered with polyethylene beads to prevent rapid water evaporation. After germination, three seedlings were retained per pot. After 7 weeks, six independent wheat rhizosphere soils were pooled for each of two soil types (S1 and S2). Five grams of each soil was cross-linked by resuspension with 2 ml of 1% formaldehyde in distilled water. The tubes were incubated at room temperature for 20 min with periodic mixing or vortexing. The samples were quenched with glycine powder (1 g/100 ml). After quenching, the tubes were further incubated at room temperature for 15 min with periodic mixing or vortexing. Finally, the cross-linked soil samples were subjected to centrifugation at 10000 x g for one min followed by rinsing with water, further centrifugation and finally the soil pellet was recovered. The pelleted samples were stored at -20°C until shipped to Phase Genomics (Seattle, WA) on dry ice. A Hi-C library was created using a ProxiMeta Hi-C Microbiome v4.0 Kit (Phase Genomics, Seattle, WA) which is the commercially available version of the Hi-C protocol. For shotgun sequencing, DNA was extracted from the same samples that were used for Hi-C using a DNeasy® PowerWater® kit (Qiagen, Venlo, Netherlands) and a metagenomic shotgun library was prepared using reagents from the ProxiMeta Kit v4.0. Shotgun sequencing was performed on an Illumina NovaSeq6000 initially to generate 100 M reads with subsequent additional sequencing to make 500 million PE150 read pairs.

### Shotgun assembly and binning

**Fig. 1:**
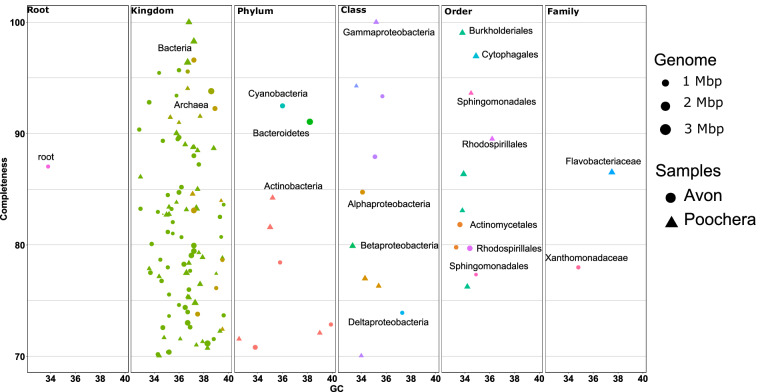
Conventional shotgun read based binning showing 134 bins with a genome completion of more than 70%. Each triangle and dot represent individual bins for Avon (S1) and Poochera (S2) soils, the size of circle and triangle depicts the genome length, Poochera (S1) and Avon (S2) bins are shown as triangles and circles, respectively. The genomes were coloured according to their marker lineage. The bins were categorized as per their marker lineage belonging to different taxa levels.

For conventional individual shotgun assembly, raw reads were quality and adapter filtered and assembled using MEGAHIT with the k-mer range set to 21,41,61,81,99 to account for sample complexity with a command line “kmin-1 pass–presets meta-large–k-list 21,41,61,81,99–no-mercy–min-count 2”^20^. For comparison of HI-C binning to the traditional shotgun binning, assembled contigs were clustered into individual bins using MaxBin 2.0^21^. The MAGs were dereplicated using dRep tool^22^. The completeness (Cp) and contamination (Ct) and marker lineage assignment of MAGs were estimated using CheckM V1.2.2^23^. These analyses were done using CSIRO high performing computing clusters. The codes for this analysis are given in “Code Availability” section. Altogether, 2,407,585,732 bp paired Illumina shotgun reads, including 68.5 and 75.4 Gb of S1 and S2 were generated from two rhizosphere soils, including 550,333,479 and 653,459,117 forward and reverse reads each in S1 and S2, respectively. After trimming, size filtering and removal of low-quality reads, we retained 59.9 Gb of reads in S1 and 67 Gb in S2 samples and used for an assembly process. The assembly of the clean 2 × 465,199,020 paired end reads generated a total of 21,975,859 contigs (334324 bp in the largest contig), with L/N50 of 557 bp in S1 while for S2 it produced 30,207,272 contigs (124,994 bp in the largest contig), with L/N50 of 564 bp from clean 2 × 517,280,905. Altogether 1,089 draft MAGs (bins) with 314 and 134 having an estimated completeness greater than 50% and 70%, respectively, were retrieved from both samples. These MAGs were associated broadly with 40 taxa, but only a few were high-quality, and most were prone to contamination (Supplementary table 1). Figure 1 describes the assembly of 134 bins with a completion of more than 70% in two different soils. Among 134 bins, 101 were associated with the taxon level kingdom belonging to two broad taxa, Bacteria and Archaea. Three taxa were identified at a phylum level, including Cyanobacteria, Bacteroidetes and Actinobacteria. At a class level, four taxa were identified between ten bins: Gammaproteobacteria, Alphaproteobacteria, Betaproteobacteria and Deltaproteobacteria. Altogether, seven taxa were distributed to 11 bins at an order level. Finally, four MAGs were classified to the family level, belonging to Flavobacteriaceae in S1 and Xanthomonadaceae in S2.

### Hybrid Hi-C assembly and binning

Shotgun reads were filtered and trimmed for quality and normalized using fastp before assembly by MEGAHIT^20^ with the k-mer range set to 25–105 to account for sample complexity, while all other parameters were set to default. Hi-C reads were then aligned to the de novo shotgun assembly according to Phase Genomics instructions https://phasegenomics.github.io/2019/09/19/hic-alignment-and-qc.html. Reads were aligned using BWA-MEM^24^ with the -5SP options specified and all other options set to default. Alignments were then filtered with samtools^25^ using the -F 2304 filtering flag to remove non-primary and secondary alignments.

**Fig. 2:**
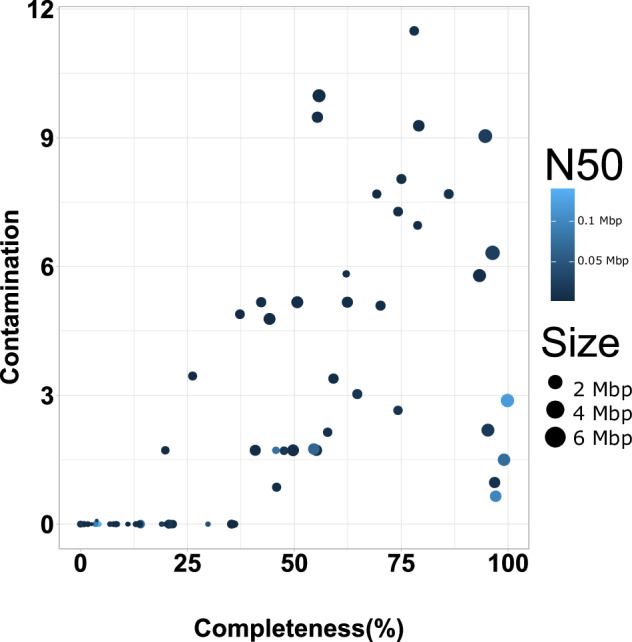
Binning information of hybrid Hi-C MAGs retrieved from Poochera (S1) soil. X- axis shows bin completeness and y-axis shows the corresponding genomes’ contamination as percentage. Each dot represents one of 88 MAGs. The colour represents the size of N50, and size of the circle represents the genome size.

**Fig. 3:**
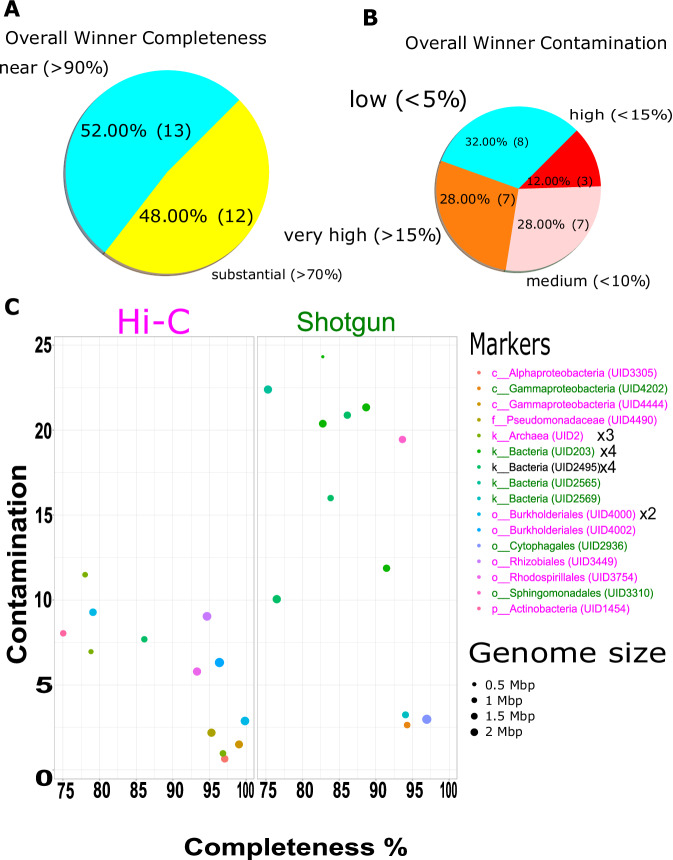
Dereplicated genomes retrieved from Poochera soil. (**A**) Pie-chart showing the percentage of bins and their completeness. (**B**) Pie-chart depicts the categorization of bins according to contamination percentage. (**C**) Shows completeness and contamination along with their genome size and marker lineage of each bin from Hi-C and Shotgun assembly. Winners are high-quality MAGs with 95% average nucleotide identity from Shotgun and Hi-C recovered MAGs analysed from a dRep program. Marker lineages with pink colour are those assigned to Hi-C MAGs and green to Shotgun MAGs. One lineage, Bacteria UID2495, was found in both data sets. The numbers alongside with the taxa name suggest number of bins belonging to the same taxa level.

**Fig. 4:**
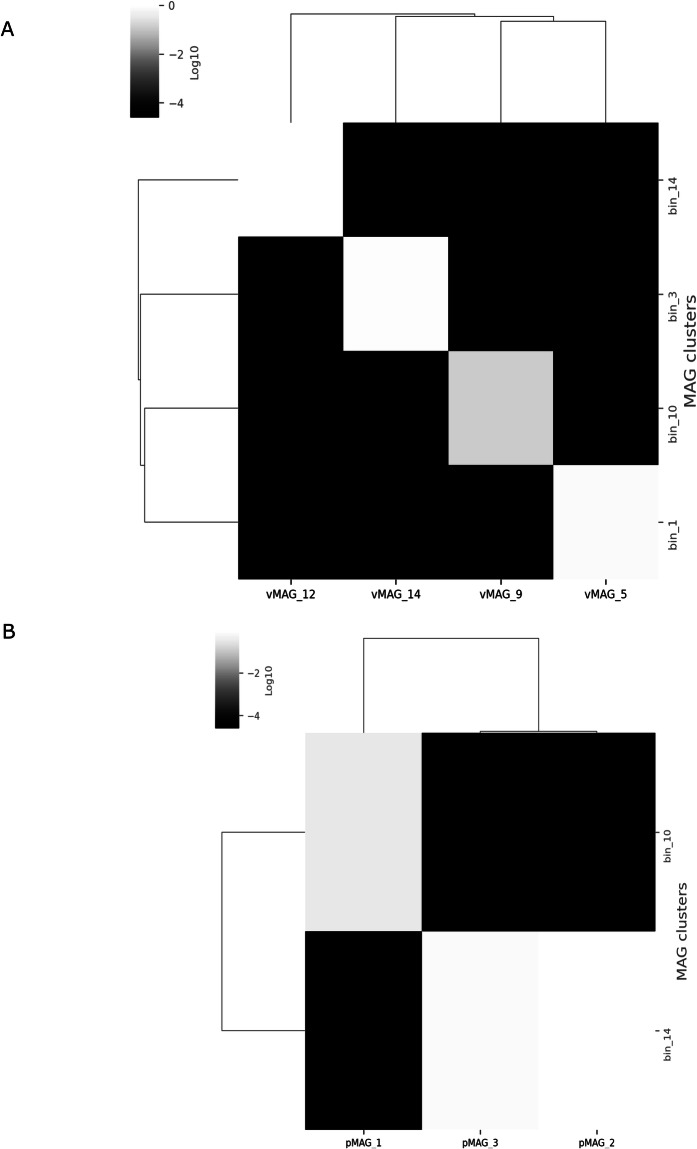
The heatmap showing the mobile element copies per cell. (**A**) Viral MAGs and (**B**) Plasmid MAGs associated with host clusters.

Deconvolution of contigs into clusters was performed using ProxiMeta as described in^26^. Briefly, contigs shorter than 1000 bp or containing fewer than two restriction sites for the enzymes used in library preparation were rejected prior to clustering. This dataset was normalized by the number of contiguous restriction sites and the contiguous Hi-C read ceiling, which indirectly accounts for contig length and abundance. Finally, the contigs were grouped into clusters/bins based on their Hi-C linkages using a proprietary Markov chain Monte Carlo method. Mobile element-host linkages were identified from Hi-C binning as described in Press *et al*.^27^. In brief, mobile element-host linkages were filtered to keep only connections with at least two Hi-C read links between the mobile element and host MAG, a connectivity ratio of 0.1, and intra-MAG connectivity of 10 links to remove false positives. For the final threshold value, a receiver operating characteristic (ROC) curve was used to determine the optimal copy count cut-off value. The optimal cut-off was determined from the ROC curve as the value that produces the point to the top left of the plot, or the cut-off that removed the maximum number of mobile element-host links while still identifying at least one host for the maximum number of mobile elements. All these analyses were performed using proprietary pipelines from Phase Genomics. Table 1 shows Hi-C bin information for two soil types. Altogether, 88 genomic bins (MAGs) were recovered from S1 sample, however only eight were retrieved from S2 with only two bins with more than 50% completion, suggesting Hi-C binning was not successful for S2 sample. The lower number of MAGs assembled using the S1 Hi-C data (88 MAGs) compared to MAGs assembled using shotgun reads only (536) reflects the more complete and higher quality Hi-C MAGs. This is further supported by evidence from dereplication of combined Hi-C and shotgun MAGs, where higher quality MAGs were retained from hybrid Hi-C binning (discussed below) as shown in Fig. 3. Among 88 MAGs, most were high-quality, with 29 scoring over 70% complete and less than 10% contaminated, with six of them being near-complete genomes with more than 95% completion and less than 3% contamination. The marker lineage for these six bins were Burkholderiales (2), Gammaproteobacteria, Alphaproteobacteria, Archaea and Pseudomonadales with a corresponding mash reference of *Sphaerotilus natans subsp. Natans DSM_6575, Marinospirillum minutulum DSM_6287, Sphingomonas like bacterium_B12, Cynoglossus_semilaevis, Pseudoduganella violaceinigra DSM_15887*. The details for the 88 bins are shown in supplementary Table 2, while Fig. 2 shows completeness and contamination of the genomes along with their genome size and contig N50. Analysis of infection networks comprised of mobile element and host pairs were identified for four hosts associated with viral MAGs and two hosts associated with plasmid MAGs in the S1 sample. Figure 4A and B shows the heatmap of an interaction pattern of viral and plasmid elements with their assigned hosts. MAGs identified as hosts included members of Burkholderiales (UID4002) and Bacteria (UID203). Furthermore, 10 viral contigs were predicted from MAGs of *Pseudomonas fluorescence* (UID4490). Additionally, seven AMR-like genes were associated with these contigs. Table 2 shows the details of annotated AMR-like genes along with their putative class and functions. The *P. fluorescens* (UID4490) draft genome was assembled to a completion of 95.71% with 2.39% contamination and novelty score of 74.13, which is considered as a high-quality MAG*. P. fluorescens* had an assembled genome size of 5,752,280 with a contig N50 of 51,591 bp. Dereplication of MAGs from Hi-C and shotgun at more than 95% average nucleotide identity resulted in 25 non-redundant MAGs with over 70% completeness (Fig. 3A). Thirteen MAGs had over 90% completion and 15 scored low contamination (<10%) (Fig. 3B). From the 15 low contaminated MAGs with more than 90% completion, 12 were retained from Hi-C deconvoluted genomes and only three belonged to shotgun (Fig. 3C). Altogether, 16 marker lineages were associated with these genomes. Interestingly, shotgun genomes could only be classified to higher level lineages (Kingdom) compared to Hi-C genomes where marker lineage was assigned up to family level with most of the lineages assigned to class and order. One genome from each set belonged to a taxon “bacterium (UID2495)”.

**Table 2:** Identification of antimicrobial resistance genes associated with* Pseudomonas fluorescence* (bin 6).

Contig id	Gene symbol	Sequence name	Element type	Class	Accession of closest sequence	Name of closest sequence
k141_1411175	emhC	efflux RND transporter outer membrane subunit (EmhC)	AMR	EFFLUX	AAQ92182.1	efflux RND transporter outer membrane subunit EmhC
k141_2293707	vanR-O	VanO-type vancomycin resistance response regulator transcription factor (VanR)	AMR	GLYCOPEPTIDE	WP_063856729.1	VanO-type vancomycin resistance response regulator transcription factor VanR
k141_2976538	copA	laccase-like oxidase (CopA)	STRESS	COPPER	AAP88295.1	laccase-like oxidase CopA
k141_3491678	vanR-O	VanO-type vancomycin resistance response regulator transcription factor (VanR)	AMR	GLYCOPEPTIDE	WP_063856729.1	VanO-type vancomycin resistance response regulator transcription factor VanR
k141_4597424	emhA	efflux RND transporter periplasmic adaptor subunit (EmhA)	AMR	EFFLUX	AAY90617.2	efflux RND transporter periplasmic adaptor subunit EmhA
k141_474788	catB1	type B-1 chloramphenicol O-acetyltransferase (CatB1)	AMR	PHENICOL	WP_010974114.1	type B-1 chloramphenicol O-acetyltransferase CatB1
k141_4829099	emhB	efflux RND transporter permease subunit (EmhB)	AMR	EFFLUX	ACO06752.1	efflux RND transporter permease subunit EmhB
k141_6068478	vanR-O	VanO-type vancomycin resistance response regulator transcription factor (VanR)	AMR	GLYCOPEPTIDE	WP_063856729.1	VanO-type vancomycin resistance response regulator transcription factor VanR
k141_615145	vanR-O	VanO-type vancomycin resistance response regulator transcription factor (VanR)	AMR	GLYCOPEPTIDE	WP_063856729.1	VanO-type vancomycin resistance response regulator transcription factor VanR
k141_6832220	cueA	copper resistance metal-translocating P1-type ATPase (CueA)	STRESS	COPPER	AAM88668.1	copper resistance metal-translocating P1-type ATPase CueA

## Data Records

The raw shotgun and Hi-C fastq read were deposited under Bio Project Id “PRJNA1126017” under following SRA accession numbers.

The Hi-C sequencing data for S1 sample were deposited in the Sequence Read Archive at the NCBI SRR29488226^28^.

The Hi-C sequencing data for S2 sample were deposited in the Sequence Read Archive at the NCBI SRR30694476^29^.

The Shotgun sequencing data for S1 sample were deposited in the Sequence Read Archive at the NCBI SRR29488227^30^.

The Shotgun sequencing data for S2 sample were deposited in the Sequencing Read Archive at the NCBI SRR29488225^31^.

The assembled Hi-C and Shotgun based MAGs were deposited in the Sequencing Read Archive at the NCBI Bio project PRJNA1126017^32^. The accession associated with MAGs are available in the supplementary table 3.

## Technical Validation

Quality control of all sequencing reads was conducted in terms of sample quality and library preparation. To ensure the quality of the Hi-C library, we first skim-sequenced the library on an iSeq instrument. The reads were then aligned to the metagenome assembly using BWA with the -5SP flag. We utilized the hic-qc tool (https://github.com/phasegenomics/hic_qc) to perform thorough quality checks on the library, ensuring the integrity and reliability of the sequencing data.

## Supplementary information

Supplementary Table 1

Supplementary Table 2

Supplementary Table 3

## Data Availability

**Bbmap:Adapter trimming and filtering** bbduk.sh in1 = S1_ShotgunFp.fastq.gz in2 = S1_ShotgunRp.fastq.gz out1 = S1_Shotgun_bbduk_1.fastq.gz out2 = S1_Shotgun_bbduk_2.fastq.gz ktrim = r k = 23 mink = 11 hdist = 1 tbo = t qtrim = r trimq = 20 ref = adapter.fa **Bbmap: Mapping of fastq to assembled contigs to generate an abundance file** bbmap.sh in1 = S1_Shotgun_bbduk_1.fastq.gz in2 = S1_Shotgun_bbduk_2.fastq.gz out = S1.sam ref = $ref ambig = all vslow maxsites = 1000 **Generation of abundance file from a mapped sam file** pileup.sh in = S1.sam out = covdepth.txt awk ‘{print $1”\t”$5}’ covdepth.txt | grep -v ‘^#‘ > abundance.txt **Megahit for metagenomes assembly** megahit -1 S1_Shotgun_bbduk_1.fastq.gz -2 S1_Shotgun_bbduk_2.fastq.gz -t -o megahitS1–kmin-1pass–presets meta-large–k-list 21,41,61,81,99–no-mercy–min-count 2 **Maxbin for binning metagenomes assembled contigs** run_MaxBin.pl -thread 64 -max_iteration 30 -contig final.contigs.fa -out maxbinS1 -abund abundance.txt **dRep for genome comparison** dRep dereplicate outout_directory -g maxbinS1/*.fasta
